# Modeling clinical activities based on multi-perspective declarative process mining with openEHR’s characteristic

**DOI:** 10.1186/s12911-020-01323-7

**Published:** 2020-12-15

**Authors:** Haifeng Xu, Jianfei Pang, Xi Yang, Jinghui Yu, Xuemeng Li, Dongsheng Zhao

**Affiliations:** 1 Information Center, Academy of Military Medical Sciences, Beijing, China; 2 Medical Service Department, General Hospital of Xinjiang Military Command, Urumchi, China

**Keywords:** Clinical events, Declarative modeling, Multi-perspective, openEHR, Process mining

## Abstract

**Background:**

It is significant to model clinical activities for process mining, which assists in improving medical service quality. However, current process mining studies in healthcare pay more attention to the control flow of events, while the data properties and the time perspective are generally ignored. Moreover, classifying event attributes from the view of computers usually are difficult for medical experts. There are also problems of model sharing and reusing after it is generated.

**Methods:**

In this paper, we presented a constraint-based method using multi-perspective declarative process mining, supporting healthcare personnel to model clinical processes by themselves. Inspired by openEHR, we classified event attributes into seven types, and each relationship between these types is represented in a Constrained Relationship Matrix. Finally, a conformance checking algorithm is designed.

**Results:**

The method was verified in a retrospective observational case study, which consists of Electronic Medical Record (EMR) of 358 patients from a large general hospital in China. We take the ischemic stroke treatment process as an example to check compliance with clinical guidelines. Conformance checking results are analyzed and confirmed by medical experts.

**Conclusions:**

This representation approach was applicable with the characteristic of easily understandable and expandable for modeling clinical activities, supporting to share the models created across different medical facilities.

## Background

The quality of healthcare is often determined by the effectiveness of medical process, which is comprised of numerous activities, such as prevention, diagnosis, therapy and rehabilitation. Besides, the clinical process is becoming more and more complicated with the rapid advancement of medical techniques. Furthermore, both reducing healthcare costs and increasing patient satisfaction requires using resources efficiently and transparently executing processes [1]. Nevertheless, traditional process improvement methods such as questionnaires and interviews may be affected by participants’ subjectivity [2]. At present, medical quality control strategies usually emphasize the Key Performance Indicator of treatment outcomes (e.g., length of stay, cure rate) than the clinical process. Since these statistical based indicators are calculated after patients leaving hospitals, they have limitations of lag and inability to find latent adverse events.

With being widely used in clinic business, information systems have generated and recorded abundant event logs across diagnosis and treatment processes [3]. An event log consists of properties related to each process instance, such as timestamp, activity, resource, etc. Process mining focuses on event logs analysis, and it can be used to three kinds of application: i) discovery process models, ii) conformance checking with predefined or discovered process models, iii) enhance or extend existing process models with the information recorded in event data [4]. For the first type of process mining, there are many algorithms which take event logs as input, and discover process models without referring any other prior information [5, 6]. After the process model is created, conformance checking (the second type of process mining) compares it with actual execution traces, shows where the actual activities deviate from the process models, or quantifies the level of compliance [7]. Enhancement is the third type of process mining, aiming to improve current process models with information such as decision points or bottlenecks.

It is necessary to represent clinical activities in modeling languages so as to process analysis and mining. At present, there are two classes of business process modeling languages: imperative approaches and descriptive approaches [8]. Imperative approaches (e.g., Petri-net and BPMN) specify the permitted event traces explicitly [8]. Nevertheless, due to the particularity of patients being treated, clinical processes usually are flexible and complex [9, 10]. Accordingly, we should support process models in healthcare in a more flexible way rather than limit users to take suitable actions [11]. To achieve this goal, descriptive approaches (e.g., Declare) only specify what activities should be done without strictly enforcing their orders for completion. Using constraint to restrict the relationship between activities, descriptive process modeling language is more adapted for describing clinical business. The scope of constraints ranges from classical sequence pattern to loosen relations, prohibitions and cardinality constraints [12]. Furthermore, most current process mining approaches in healthcare generally pay attention to the control flow of activities. In contrast, the perspective of data flow and the time properties are ignored in general [13]. In order to comprehensively model and compliance check for medical processes, it is necessary to consider these different perspectives fully.

At present, clinical processes modeling (by handmade or discovered automatically) is predominantly carried out by information technology (IT) staff. Nevertheless, they often have to spend a long time to consult with medical specialists for clinical knowledge or therapy procedures, and it is also difficult to ensure the modeling effect. Modeling guided by medical professionals may provide an alternative solution, because they really understand their data and workflows. However, classifying event attributes from the view of computers is difficult for medical experts. IT personnel should support the healthcare experts in the conformance evaluation tasks, supplying them with semantic modeling tools, and reducing the difficulty to model medical process [14].

At the same time, generating each process model requires a certain cost, and every model designer may have a different understanding of business rules. Therefore, we should ensure the stability of established models, and address the problems of sharing and reusing in different institutions after they are generated [15, 16]. To achieve this target, openEHR published the technical specifications such as prototypes and templates, which defined the platform and the clinical domain models. However, it needs further consideration of process and workflow aspects in the openEHR specifications [17].

To address these problems, we presented a constraint-based method using multi-perspective declarative process mining, to model clinical activities with openEHR’s characteristics. It integrates the representation of control flow and data flow, including resource and time properties, supporting medical professionals to model clinical processes without IT personnel deeply involved. Moreover, this method is easy for extending new medical activities into current attribute types. Finally, a real-life data set is used to evaluate our approach with preliminary results.

This paper is an extension of work previously published at the Biomedical and Health Informatics (BHI) workshop of 2019 IEEE International Conferences on Bioinformatics and Biomedicine (BIBM) [7]. In this extension, we give a more detailed description of the idea and offer a complete algorithm of conformance checking, adding a preliminary knowledge section as well. We also expand the case study with additional data for experiment, and add a discussion section to compare our method with other related works.

## Preliminary knowledge

### Event log

**Fig. 1:**
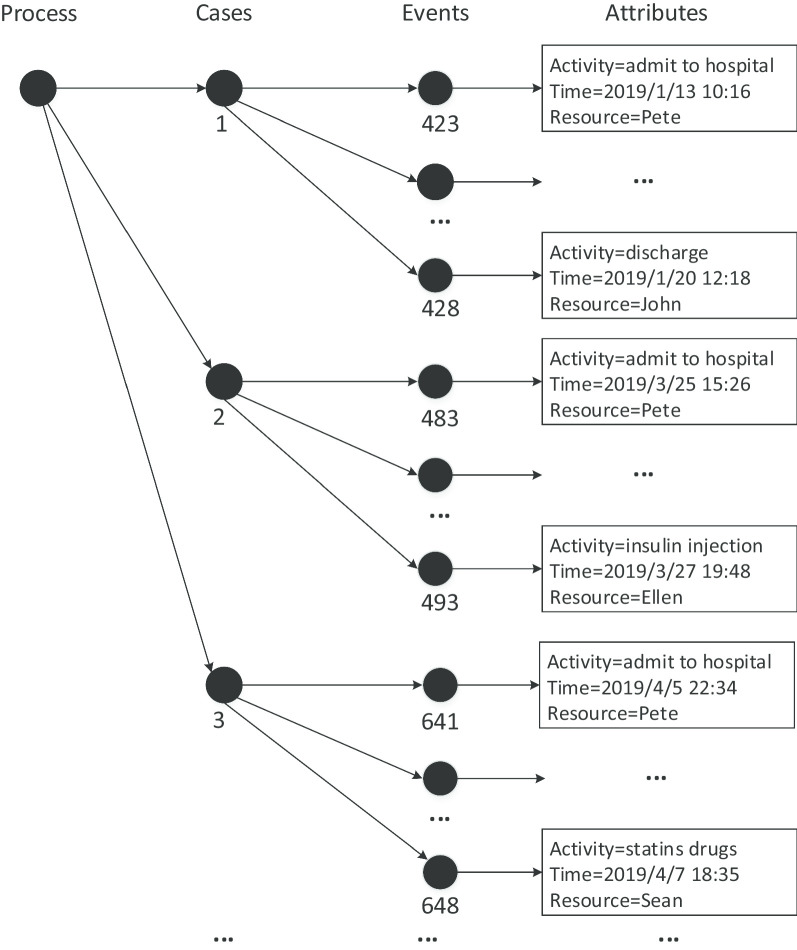
The structure of an event log

Event logs provide detailed information as the basis of process mining and analyzing. Table 1 illustrates a part of event logs recorded in the ischemic stroke treatment process. Every row represents an event, and each event could contain a number of process-related information, such as activity name, resource (actors), timestamp, etc. The lifecycle of an event includes schedule, start, complete, and so on. The tree structure of an event log is illustrated in Fig. 1. Each process may consist of multiple cases; each case can contain a number of events, while every event can only belong to a single case; events in each case are arranged orderly, and each event may have multiple attributes [4]. XES (eXtensible Event Stream) is a structural specification of data files used to store, exchange and analyze event logs. It was accepted by the IEEE Task Force on Process Mining, and has been widely supported by many process mining software like ProM and Disco [4]. An XES log file may contain any number of traces, and each trace, with multiple attributes, is a finite event sequence. The constraints between events could be equivalent to the constraints between attributes of events. XES's metadata model provides five types of event attributes: Boolean, Float, Integer, String and Date. However, these attributes are from the perspective of computer science to classify data without describing specific medical semantics, which is adverse to the interpretation and application of healthcare experts for process modeling.

**Table 1:** A part of event logs in healthcare

Case_ID	Event_ID	Properties			
		Activity	Actor	Timestamp	…
1	423	Admit	Pete	2019/1/13 10:16	…
1	424	CT examination	Sue	2019/1/13 10:26	…
1	425	Blood sugar test	Mike	2019/1/13 10:19	…
1	426	Intravenous thrombolysis	Sara	2019/1/13 10:56	…
1	427	Statins drugs	Mike	2019/1/13 11:38	…
1	428	Discharge	John	2019/1/20 12:18	…
2	483	Admit	Pete	2019/3/25 15:16	…
2	485	Blood sugar test	Mike	2019/3/25 15:26	…
2	487	MRI examination	Sue	2019/3/25 15:45	…
2	488	Have a fever	Sara	2019/3/26 08:30	…
2	489	Anticoagulant drugs	Sean	2019/3/26 11:16	…
2	490	Antiplatelet drugs	Mike	2019/3/27 10:22	…
2	493	Insulin injection	Ellen	2019/3/27 19:48	…
3	641	Admit	Pete	2019/4/5 22:34	…
3	643	Intravenous thrombolysis	Sara	2019/4/5 23:46	…
3	644	Hypertensive	Mike	2019/4/6 09:10	…
3	645	Anticoagulant drugs	Sean	2019/4/6 13:10	…
3	648	Statins drugs	Sean	2019/4/7 18:35	…
…	…	…	…	…	…

### openEHR

To date, studies for process mining in healthcare are mostly led by medical informatics researchers and computer scientists. However, due to the specialization of medical knowledge and the complexity of medical process, clinical experts should play a dominant role in medical process modeling. This kind of modeling idea is advocated by OpenEHR, an international standard of ISO for information modeling in medical domain. It has a two-layer model architecture composed of a reference model and archetype, which assures the stability of the underlying organization of software and increases the adaptability of the system to change with domain knowledge [15]. Medical process consists of clinical-related process (e.g., diagnosis and treatment) and organizational management process (e.g., registration and admission). openEHR community has designed a technology ecosystem including domain modeling formalism, terminology and factory environment, in order to fulfill the need for modeling clinical and management processes. By sharing archetypes and templates developed by domain professionals, different medical institutions could achieve interoperability in accordance with the standard of openEHR. Each archetype is the largest data set involved in an independent clinical activity, whereas the template suits for specific applications to generate clinical models by adding constraints to archetype [15].

The archetypes of openEHR could be classified into five categories: Admin, Observation, Evaluation, Instruction and Action. The Admin entry represents clinical management procedures, such as admission, discharge, transfer and so on. The other four categories are designed according to clinical diagnosis and treatment process. For example, Observation entry is an objective measure to depict the symptoms of patients by clinical staff, such as body temperature or blood pressure; Evaluation entry is the preliminary diagnosis and assessment of a patient's health status based on the results of observation, medical knowledge and clinical evidence; Instruction entry is a treatment plan proposed by a doctor for the patient, such as prescribing, examine and testing application forms; Action entry is to intervene or treat patients according to the treatment plan ordered by doctors, such as drug administration and blood matching. In addition, openEHR offers a demographic archetype, which contains essential information about people’s privacy data, such as patient name and address. The demographic archetype contains two subclasses: actor and role. The actor refers to the types of individuals and organizations in the real world, while the role represents the part which actors can play.

### Declarative process mining

In the field of medical informatics, the application of process mining is primarily for discovering process models, checking conformance, and enhancement. Traditional process modeling approaches, which have been used by most process modeling languages, are essentially imperative, and they prescribe how to work strictly with less semantics [8]. For example, there are only four kinds of relationships between activities in Petri-net [4].

- Direct succession: x > y iff for some case x is directly followed by y;
- Causality: x- > y iff x > y and not y > x;
- Parallel: x||y iff x > y and y > x;
- Choice: x#y iff not x > y and not y > x;

However, in many medical circumstances, a physician needs to address patients in a flexible way depending on their condition. Furthermore, the generated process models using imperative approaches often have to be changed frequently with medical progress [18]. On the contrary, declarative modeling approaches predefine what needs to be done without prescribing how to work. They provide a wide variety of relations, called constraints, to represent rules that should be followed, and any behavior that does not violate them is acceptable. Linear temporal logic (LTL) could be applied to define constraints of events for declarative languages [8]. LTL also contains temporal operators, for example, always (□F), eventually (◊F), next time (○F), until (F⊔G) and weak until (W), besides the basic logic operators. Nevertheless, it is hard to understand LTL formulas by non-computer experts due to the complicated expressions.

**Fig. 2:**
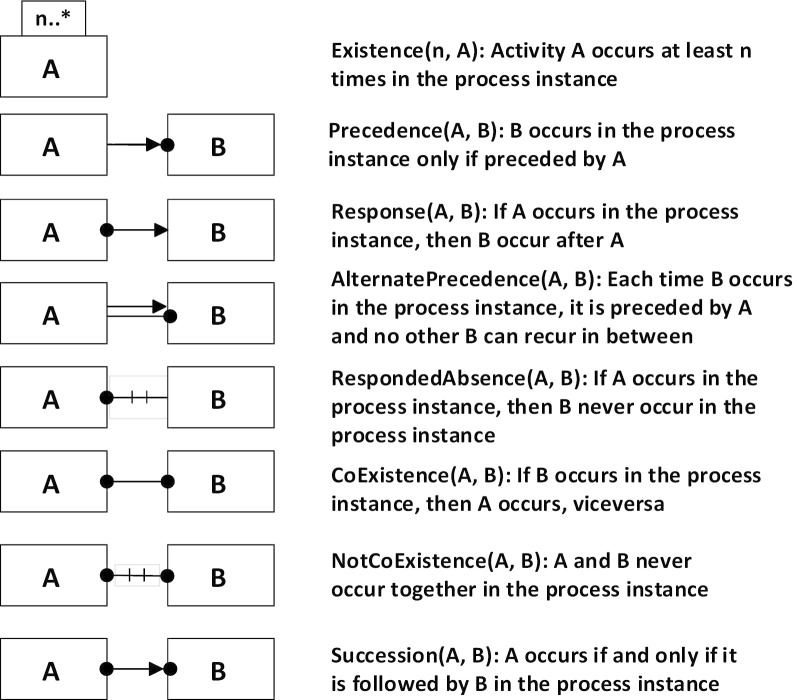
Examples of constraint templates

Based on finite traces, Declare [18] defines templates of constraints using LTL, and integrates graphical representation with each template. There are four kinds of templates: Existence, Relation, Negative Relation and Choice. For example, the Relation template outlines relationships like Precedence, Response, Alternate Precedence, and so on for activities. Some examples of declarative templates are shown in Fig. 2. In order to supports flexibility, Declare provides mandatory as well as optional constraints. The optional constraints may be violated without an alarm, while the mandatory constraints cannot be violated. Besides, the set of Declare templates are extendible, and it works best with flexible processes, so we choose to extend Declare for modeling clinical activities.

## Methods

As computer technology is becoming a universal tool, clinical experts need methods that can facilitate the modeling of medical process. IT personnel should provide technology and tools to assist this work. We refer to the classification ideology of archetypes in openEHR for medical activities, so as to enable clinical experts to understand the attributes and relationships of events clearly and intuitively. The event attributes are classified based on medical services, and each constraint between events is expressed in a relational matrix, which can be extended and understood by clinical experts easily. Moreover, in order to model and represent the medical process comprehensively, based on the traditional control flow model, the data constraints between events are added, which can represent the multi-perspective relations such as time flow, organization flow and so on.

### Representation Method

**Fig. 3:**
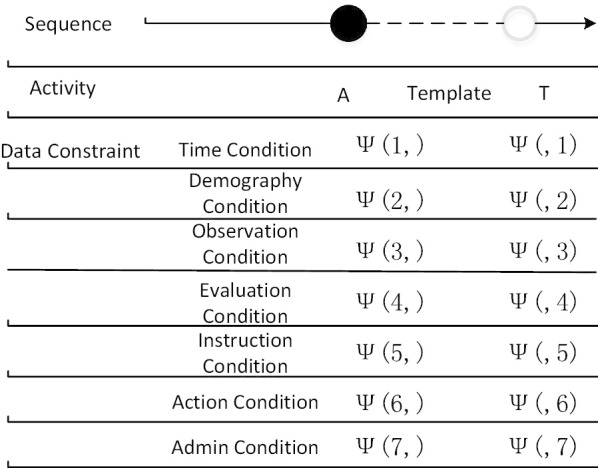
Model composition diagram

The non-atomic activities (start and complete in particular), the resource who originated event (originator), the metric temporal restrictions for which event should be completed, cannot be represented sufficiently by the standard LTL meanings of Declare [12]. Enlightened by [13], to realize computer understandable event log checking, we expand Declare language and design a constraint-based approach for process representation. Figure 3 illustrates the model composition diagram. This representation method not only can specify the control flow between events, but also the data flow information such as resource (organization), time, etc. Given the set of all possible activities Ĕ within a procedure, we define a constraint as a tuple: c = < Type, A, T, ψ > , where Type indicates which template the constraint is referring to, Type ∈ {existence, absence, choice, responded existence,...}; A is a source event and A ∈ Ĕ; T is a target event and T ∈ Ĕ, A ∩ T ≠ NULL; ψ denotes the data condition. There are three subclasses of ψ: ψ(i,) is the active condition on A; ψ(,j) represents the data condition on T; and ψ(i, j) indicates the restraint condition between A and T. Each data condition ψ is a predicate or a formula Expr1 Op Expr2 where Op ∈ {= , ≠ , < , > , ≤ , ≥}, and Expr1, Expr2 are constituted by data identifiers or constants (numerical or string). We classify constraints into two categories: unary constraints include the Existence and Choice of Declare, and binary constraints contain the Relation and Negation Relation of Declare. It should be noted that one rule from clinical guidelines written in natural language might be transformed into multiple ψ or constraints.

**Fig. 4:**
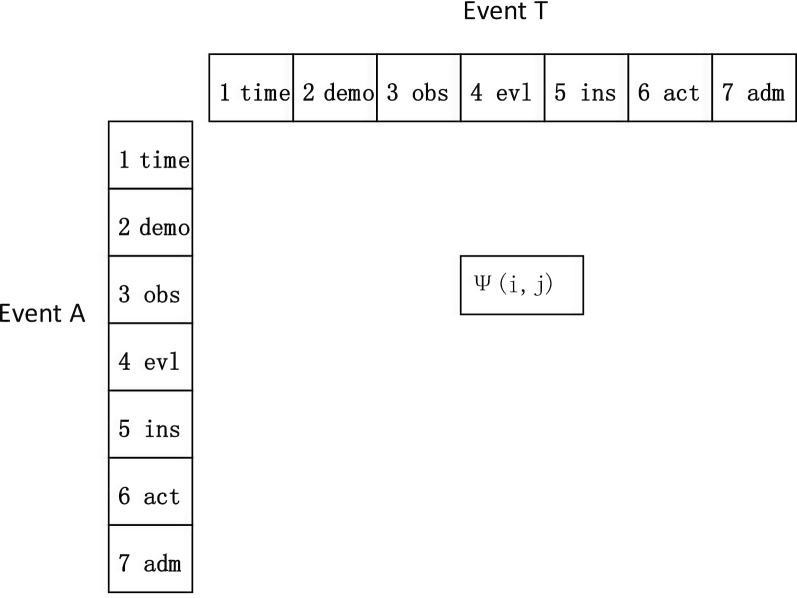
Constraint Relation Matrix

Seven kinds of event attributes are defined in this study: time, demography, observation, evaluation, instruction, action and admin. The time attributes comprise information of lifecycle states of each activity, for example, starting time, completion time, etc. The other attributes are in line with the meanings of openEHR’s archetypes. For data flow constraints, all of the event attributes between a source event and a target event constitute a Constrained Relationship Matrix of <A, T > (Fig. 4). Each ψ(i, j) specifies a unique constraint between event attributes, which is NULL if there are no constraints between the two event attributes. This representation method can be applied in both the automatic (or handmade) discovery model and the compliance checking algorithm. After compliance checking on a specific event log, the overall evaluating result for every constraint C contains two sections: control flow checking result and data flow checking result. One constraint C between event A and T will be unsatisfied if either result of the section is unsatisfied, or else the constraint C is satisfied.

### Conformance checking algorithm

Conformance checking on event logs with process models can audit the actual implementation of guidelines, or assess the quality of models discovered (or handmade) to provide information for process improvement [18]. We propose a checking algorithm in Algorithm 1. Taking a trace and a constraint C as input, our algorithm could generate the set of violated and fulfilled events in that trace. All the events are iterated for each trace: firstly, checking whether the attribute of event A has satisfied the activation condition prescribed in template(t) and ψ(i,); secondly, verifying whether A in the pending and corresponding T satisfy the template condition and the data constraint ψ(i, j), that is whether the corresponding variables in the ith attribute of event A and the jth attribute of event T agree with ψ(i, j); thirdly, checking whether the attribute of T satisfies the data condition specified in ψ(, j); lastly, if neither the template condition nor the data constraint ψ(i, j) is violated, adding e to the fulfillments set; otherwise, adding event e to the violations set.
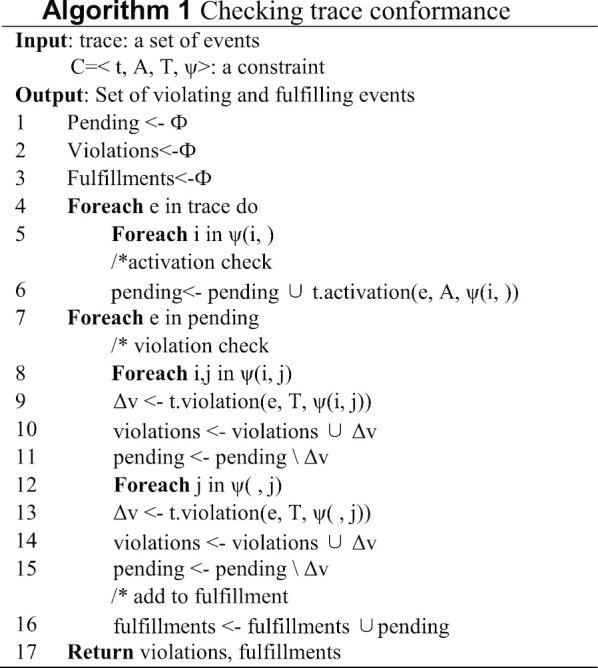


## Results

Stroke is one of the most important factors for the disability and death of adults in the world, which has caused a heavy economic burden, according to the World Health Organization [19]. We choose the therapy process for ischemic stroke, which makes up almost 70% of all stroke, as a case study. Although there are some algorithms supporting to discover process models automatically based on event logs, it remains necessary to revise and validate these models manually by experts. In this research, we established a group consist of three medical specialists, three informatics experts and one database engineer, to produce a handmade model applying the method proposed.

### Manual modeling steps

**Fig. 5:**
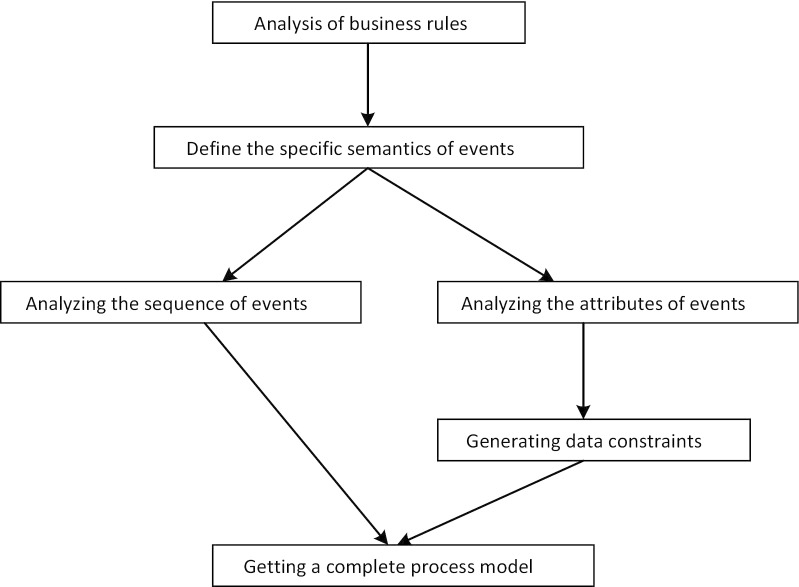
Over all steps of manually modelling

We show the complete manual modeling steps of this experiment in Fig. 5. Firstly, based on the medical problems to be solved, relevant standard documents as well as business rules, are searched and discussed. Secondly, determining the medical events involved and the specific semantics of each event corresponding to the data set in production systems. After the control flow of events is ascertained, the attribute types of events are analyzed from the point of view in medical services, and the data constraints among these attributes are determined. Finally, a complete process model is generated.

### Constraint-based process model

Depending on the questions considered for clinical quality management, informatics specialists of our group chose five typical rules in stroke diagnosis and treatment process. Table 2 lists examples of the process model generated, including sequence, time, personnel and other factors. At present, the most effective strategy for acute ischemic stroke patients, is to inject thrombolytic medicine for reperfusion as soon as possible. The time from onset to thrombolysis has a significant impact on the success of rescue. In China, there are two commonly used thrombolytic medicine: recombinant tissue-type plasminogen activator (rt-PA) and urokinase. The door-to-needle time (DNT) is recommended no more than 60 min for therapy of ischemic stroke in clinical guidelines (CG) [20]. Moreover, it is necessary to test CT (or MRI) and blood glucose previous to intravenous (IV) thrombolysis, and blood pressure (BP) need to be kept less than 180/100 mm Hg. In addition, if patients have received a treatment dose of low-molecular-weight heparin within the previous 24 h, IV alteplase should not be carried out. Lastly, so as to avoid adverse medical events, it is suggested that at least two nurses execute a double-checking of the dispensing drugs [21].

**Table 2:** Part of process models for stroke diagnosis and treatment

No	Rule description	Constraint C	Data constraints
1	CT and blood glucose should be tested before IV thrombolysis	< precedence, CT examination, thrombolysis, > and < precedence, blood glucose test, thrombolysis, >	
2	The door-to-needle time (DNT) should be within 60 min	< precedence, admit, thrombolysis, ψ(1,1) >	ψ(1,1) = [complete time, complete time,60, <]
3	BP need to be kept less than 180/100 mm Hg	< precedence, vital_signs test, antipyretic, ψ(3,) >	ψ(3,) = [systolic pressure, 180, <]&[diastolic pressure, 100, <]
4	For patients who have received a treatment dose of low-molecular-weight heparin within 24 the previous hours, IV alteplase should not be carried out	< Not coexistence, heparin injection, thrombolysis, > or < precedence, heparin injection, thrombolysis, ψ(1,1)>	ψ(1,1) = [complete time, start time, 24*60, >]
5	For dispensing drugs, at least two nurses should execute a double check	<existence, dispensing drugs, ψ(6,6)>	ψ(6,6) = [executor, proofreader, ! =]

### Checking results

We build a data set of 358 patients diagnosed with ischemic stroke from one tertiary hospital information system in China. They received a remedy of rt-PA or urokinase (281 and 77 respectively) during hospitalization from July 2009 to July 2019. We check the conformance of actual events with the process model generated above, after data preprocessing and transforming into event logs. Based on the Declare Analyzer [22] plugin of ProM, we implemented the above model in Java language. Table 3 lists the checking results of this experiment, where we use the abbreviations “vio” and “ful” to represent violation and fulfillment respectively. After sufficient discussion, the consensus of our expert team is used as a gold standard. Take rt-PA as an example, one patient received thrombolysis did not exam CT (or MRI), and 17 patients did not test blood glucose. There are 69 patients’ DNT longer than 60 min, and two patients got a heparin remedy within 24 h. The systolic blood pressure of 16 patients was larger than 180 mm Hg, while the diastolic blood pressure of 40 patients was larger than 100 mm Hg. Though the specialists in our team have validated these results using SQL (Structure Query Language) script, the reason for these variations could be various, such as the inconsistent standards of data sources and the dynamicity of patient’s condition. As a consequence, we need to further analyze these results combining with more clinical science, because the cause of variations might impact the toughness of conclusions.

**Table 3:** Checking results for ischemic stroke therapy

No	Description	rt-PA		Urokinase	
		Vio	Ful	Vio	Ful
1.1	Before IV thrombolysis, CT should be tested	1	280	0	77
1.2	Before IV thrombolysis, blood glucose should be tested	17	264	1	76
2	DNT < 60 min	69	212	27	50
3.1	Systolic < 180 mm Hg	16	265	6	71
3.2	Diastolic < 100 mm Hg	40	241	15	62
4	Dispensing drugs should be double checked by different nurses	0	281	0	77
5	Not received a treatment dose of low-molecular-weight heparin within 24 h	2	279	11	66

In spite of CGs aim to provide the best practice based on clinical evidence, which is a kind of statistical knowledge in nature, CGs represent the universality of patient population, instead of the particularity of individual. Therefore, when experts compiling a CG, they usually assume there are ideal patients, ideal physicians, and ideal execution context [23]. Nevertheless, the patient or the context might not be ideal when doctors apply a published CG to a unique patient. So as to exploit the generic CGs to a particular patient in reality, the doctors have to make use of their basic medical knowledge. Accordingly, we should neither interpret the recommendations described in CGs as “must-do” actions, nor analyze their complex semantics separating from basic medical knowledge. On the other hand, deviations from CGs might occur unavoidably and meet individual patient’s needs better. However, these variances may also decrease medical quality. Thus, we need to systematically measure and record the deviations for analysis, to provide this information for medical quality control department to evaluate the rationality of variance and the compliance of CGs.

## Discussion

In [12], the authors extended Declare constraints to tackle data-flow aspects, using the Event Calculus (EC) formalization. A similar rule-based approach is proposed in [24], where the ConDec language was extended with a Computational Logic framework to specify declarative interaction models. Awad et al. presented an extension version of BPMN-Q language [25], incorporating data perspective into the specification of rules to evaluate compliance. The authors in [26] proposed an extended Timed Declare process models, using timed automata to provide a priori guidance. However, these researches did not classify variables or attributes from the point of view in medical business, with less comprehensibility for clinical staff. Rovani et al. [11] reported a declarative process modeling language to check the compliance of CG against event logs reflecting the actual clinical practice. In [17], clinical decision support (CDS) content was expressed in the Guideline Definition Language (GDL) using openEHR archetypes and terminology bindings, supporting to check the compliance of rules prescribed in CG for acute stroke care. However, it is still uncertain whether the same GDL rules could be applied in at-the-point-of-care CDS [17].

In the community of medical informatics, there are some studies on the expression of decision-making behavior in diagnosis and therapy, for instance, the construction of Computer-Interpretable Guidelines (CIG) models. CIG mainly includes two types: Rule-based systems (‘if–then’ rules), and task network languages (TNL) such as GLIF, PROforma [27], etc. Unlike these rule-based systems, TNL organizes the control flow in a guideline model explicitly. As the number of activities increasing, Rule-based systems are limited by the ability to represent and maintain complex processes, such as clinical pathways (CP) and protocols. On the other hand, all the task network schemes represent clinical guidelines in the form of guideline plans. The plan’s components are categorized by decisions, actions, or hierarchically decomposed sub-plans of the guideline and their relationships. However, in PROforma, there is little support for modeling clinical organizations, nor for modeling communication, and all TNLs fall significantly short of coping with the set of 40 benchmark control flow patterns [28].

Compared with the hard coding method for a single task, although this method may need more steps, because a large number of modeling tasks are led by medical staff, our method is more convenient to use, and does not require IT staff to participate in the whole process. Comparing to the business modeling methods, which are not classified with openEHR’s characteristics and medical staff need to divide the attributes of events from the computer science point of view, our approach is relatively easy to be understood with medical semantics. The CG of acute ischemic stroke is relative forthright to be transferred into computer-interpretable formats, in comparison with that of other clinical diseases [17], but we believe it will not seriously damage the overall feasibility of this method. Since the models we generated are essential based on LTL expression, which has been explored and widely applied in the domain of model checking [29], our method could fully describe all kinds of possible temporal logical relations for clinical events.

The contributions of this paper are that it combines the two fields of openEHR and Process mining, supporting efficient modeling medical process in agreement with international standards. For example, although there are many perspectives in Process mining technique: control-flow, organizational, case, time, and so on, we integrate and classify them with medical expertise, which is more consistent with the usage pattern of clinicians. By converting underlying data into event logs in the data extraction step, this approach can be compatible with a variety of data storage standards.

## Conclusions

In this paper, we have introduced a modeling representation method based on multi-perspective declarative process mining, supporting medical personnel to model clinical processes with professional knowledge independently. Since we comply with openEHR specification and medical oriented classification, this approach is easy to comprehend for clinical personnel. They could classify activities and attributes in healthcare to the seven types, and transfer recommendations from CGs into constraints, for compliance checking of actual patient cases. Besides, if medical institutions classify event attributes in accordance with openEHR standards, the models generated are reusable, extensible and stable.

Since this presented method requires the name of attributes in constraints to be mapped to that in event logs, medical staff should be familiar with variable names in attribute sets to generate effective models. In the future, we intend to bind event attributes to standard terminologies, so that doctors could define quality requirements directly without knowing IT implementations. We also plan to develop algorithms for automatic discovery process models based on event logs, and for achieving conformance checking in an online manner.

## Data Availability

The dataset supporting the conclusions of this article is not available since the privacy of patients is included.

## Abbreviations

XES: EXtensible event stream
LTL: Linear temporal logic
rt-PA: Recombinant tissue-type Plasminogen Activator
CT: Computed tomography
MRI: Magnetic resonance imaging
HIS: Hospital information system
IV: Intravenous
CG: Clinical guideline
DNT: Door-to-needle time
SQL: Structure query language
BMK: Basic medical knowledge
MQC: Medical quality control
EC: Event calculus
CDS: Clinical decision support
GDL: Guideline definition language
CIG: Computer-interpretable guidelines
TNL: Task network languages
CP: Clinical pathways
